# Coarse-Grained Modelling and Temperature Effect on the Morphology of PS-*b*-PI Copolymer

**DOI:** 10.3390/polym11061008

**Published:** 2019-06-06

**Authors:** Natthiti Chiangraeng, Vannajan Sanghiran Lee, Piyarat Nimmanpipug

**Affiliations:** 1 Department of Chemistry, Faculty of Science and Center of Excellence for Innovation in Analytical Science and Technology, Chiang Mai University, Chiang Mai 50200, Thailand; natthiti.c@gmail.com; 2 Doctor of Philosophy Program in Chemistry, Faculty of Science, Chiang Mai University, Chiang Mai 50200, Thailand; 3 Department of Chemistry, Faculty of Science, University of Malaya, Kuala Lumpur 50603, Malaysia; vannajan@um.edu.my

**Keywords:** morphology, block copolymer, polystyrene, polyisoprene, phase separation

## Abstract

Spontaneous spatial organization behavior and the aggregate morphology of polystyrene-*block*-polyisoprene (PS-*b*-PI) copolymer were investigated. Molecular dynamic (MD) and mesoscopic simulations using the dynamic of mean field density functional theory (DDF) were adopted to investigate the morphology changes exhibited by this block copolymer (BCP). In the mesoscopic simulations, several atoms in repeating units were grouped together into a bead representing styrene or isoprene segments as a coarse-grained model. Inter-bead interactions and essential parameters for mesoscopic models were optimized from MD simulations. Study indicated that morphology alternations can be induced in this system at annealing temperature of 393, 493, and 533 K. From our simulations, lamellar, bicontinuous, and hexagonally packed cylindrical equilibrium morphologies were achieved. Our simulated morphologies agree well with the reported experimental evidence at the selected temperature. The process of aggregate formation and morphology evolution were concretely clarified.

## 1. Introduction

Many researchers have been attempting to understand the complexity of copolymer systems and morphological studies on these materials have played a vital role in this field [1,2,3,4]. The self-assembly of polymers is a process in which the polymeric chains spontaneously organize themselves leading to a variety of aggregated structures and morphologies. The morphology of this soft material is found to be connected to its properties as well as its applications. Block copolymers (BCPs) with specific properties have been achieved via controlled mesoscopic self-assembly. To achieve the desired applications of copolymer, morphological modification is crucially needed. A number of experimental and theoretical research studies have been dedicated to achieving this goal.

Phase segregation in BCP is driven by chemical incompatibility between the dissimilar blocks. One of the most sophisticated and simplest cases is the linear AB diblock copolymer. This copolymer consists of only two distinct monomer types. The AB diblock copolymer is appealing since it is easy to synthesize, and various morphologies can be observed due to energetically preferable arrangement. The phase diagram of linear AB diblock copolymer has been reported in a wide range of compositions versus χ*N* [5]. The χ and *N* denote the effective interaction parameter between distinct monomers and the polymerization index of a polymer, respectively.

The phase behavior of the bulk BCPs is determined by three experimentally controllable factors [6,7]: (i) The overall degree of polymerization represented as *N* (*N = N_A_ + N_B_*); (ii) the overall composition represented as *f* (*f = f_A_ + f_B_*); and (iii) the A–B segment–segment interaction parameter represented as the Flory-Huggins segment–segment interaction parameter (χ). These functional factors were concluded by Leibler since 1980 [8]. The first two factors are regulated through the study route, while the χ parameter is determined by the selection of the A-B monomer pairs. Typical morphologies for simple block copolymers (AB), which consisted of a chain of monomer A covalently bonded to a chain of monomer B, are sphere, cylinder, gyroid and lamellae [9]. Theoretical and experimental phase diagrams can be used to predict these morphologies formations. The morphology of a copolymer can be influenced by solvents [10,11,12], composition [1,13,14,15], temperature [13,15,16,17,18], external forces [19,20], additives [17,21,22,23,24,25,26], etc. Morphology of copolymer can be useful for a broad range of applications. Cylindrical and lamellar shaped copolymers can be useful for industrial purposes, e.g., PS and PMMA copolymers were used as lithographic templates [27,28,29] for nanowire construction and components of electronic devices. Lamellar, cylindrical, and gyroidal morphologies of copolymers with appropriate additives can be used in batteries and solar cells [30,31]. While the final morphology at a given condition of a copolymer can be directly identified, the experimental observation of its self-assembly behavior is far from trivial due to various factors. The copolymer morphology is one of crucial factors corresponding to applications of polymeric materials. The material, which had specific properties and morphologies, would be employed under a various conditions. Clarification of these self-assembly processes are vital for deeply understanding the morphologies and morphological-related applications of copolymers [11,32].

Many numerical algorithms have been developed for studying the morphological properties of polymer-based materials. Due to the development in the field, the morphology and phase separation mechanism of copolymeric materials can be precisely predicted and clearly described compared to the available experimental findings. For example, a lamellar structure was observed from PEO_13_PPO_30_PEO_13_ triblock copolymer in aqueous solution with high copolymer concentration [1] correlated with experimental phase diagram reported by Alexandridis et al. [33]. In addition, other three morphologies (micellar, hexagonal and bicontinuous structures) were also observed from a theoretical prediction. The dynamic of mean field density functional theory (DDF) [34,35] is one of common techniques and has been employed to study the self-assembly behavior of copolymer materials at a mesoscopic level. The mesoscopic properties of copolymers can be successfully explained and predicted by the computational simulations using these algorithms [1,36,37,38,39]. Apart from the morphology prediction, DDF can explain and observe the mechanism via trajectory analyzes [11,32] which provides some insights for better understanding the formation of copolymers and their corresponding applications.

Polystyrene-*block*-polyisoprene (PS-*b*-PI) copolymer has been selected as our model system to understand the complexity of copolymer systems on account of its complicated BCP phase behavior [40,41,42,43]. Self-assembly due to interactions in aliphatic block attached to aromatic block leads to various morphologies. Accordingly, this BCP was investigated as attributable to scientific points of view and as a candidate for use in modern applications, e.g., a smart polymer with order nanostructure. The variation of PS-*b*-PI copolymer morphologies has been comprehensively investigated in a range of compositions [5]. Several techniques have been applied both experimental and theoretical attempts [5,40,41]. In the present study, molecular dynamic (MD) and mesoscopic dynamic simulations will be carried out to represent the ability of the diblock copolymer chains to undergo aggregation. Gaussian chain parameters and interaction parameters governing the self-assembling phenomenon of PS-*b*-PI copolymer will be optimised to accomplish understanding of this microphase separation into various morphologies.

## 2. Methods

### 2.1. Molecular Dynamic Simulation

Initially, a minimum chain length of repeating unit of each polymer (MCL), which can be efficiently represented an actual polymer chain in each condition was explored. Polymers with different chain lengths, including 10, 20, 30, 40, 50, 60, 70, 80, 90, and 100 units, were constructed as amorphous structure in a cubic simulation box. Experimental densities of 1.05 and 0.90 g cm^−3^ were set for construction of PS and PI models, respectively [5]. Polymer chains were put into a simulation box. The condensed phase optimized molecular potentials for the atomistic simulation studies (COMPASS) force field [44,45] was used in this MD simulation. It is an efficiency force field suggested for organic inorganic and polymer materials. The system was minimized using a conjugate gradient method with convergence threshold of 0.01 kcal/mol/Å and energy convergence of 1.0 × 10^−6^ kcal/mol as a criterion of structural minimization. After that, an optimized amorphous structure was refined under a canonical ensemble simulation with constant number of particles, volume, and temperature (NVT ensemble) for 200 ps. A time step of 1 fs was used for MD simulation to ensure the stability of simulation. Temperature was controlled by Anderson thermostat in NVT-MD simulation. Then, the model was annealed at temperature range from its glass transition temperature ($T_{g}$) and melting temperature ($T_{m}$). Temperature ranges from 383 K to 433 K and 225 K to 285 K were used in an annealing process for PS and PI systems, respectively [46]. Annealing temperature increments of 10 K were used for 25 heating-cooling cycles. Then, a structure was refined under isothermal-isobaric ensemble simulation with constant number of particles, pressure, and temperature (NPT ensemble) for 200 ps. Anderson thermostat with a collision ratio constant of 1.0 and Barendsen barostat with a decay constant of 0.1 ps were applied to control a temperature and pressure, respectively. The annealing-refining process was repeatedly performed for four times. Finally, NVT-MD simulations were performed for 200 ps to achieve the most stable structure. In this work, the diblock copolymer of PS and PI consisted of 138 and 375 units was studied [5]. The solubility parameter of pure PS, pure PI, and mixing systems were analyzed from NVT-MD trajectories. These values were used to calculate an effective *χ* parameter. The effective *χ* parameter was subsequently used for calculating MesoDyn input parameter.

### 2.2. Mesoscopic Dynamic Simulation

Mesoscopic dynamic (MesoDyn) simulation based on DDF is one of the powerful techniques for studying polymeric morphology and its phase separation dynamic. MesoDyn treated polymer chains were investigated via the coarse-grained model. The several atoms or repeating units are grouped together into a bead with concerning its characteristic properties. Its dynamics were described by functional Langevin equations [16]. A bead diffusion coefficient was set as $1.0\times 10^{-7}\;{\mathrm{cm}}^{2}s^{-1}$. The noise parameter of 75.002 was used for controlling numerical speed and stability of system. Simulations at three different temperatures, 393, 493, and 553 K, were performed. All simulations were performed in cubic simulation box with a dimension of 32 × 32 × 32 nm^3^. The grid parameter was set as 1.0 nm and the bond length was 1.1543 nm. The total simulation time was 10 ms with the time step of 50 ns.

## 3. Results and Discussion

### 3.1. Variation of Cohesive Energy Density and Gaussian Chain Model Development

**Figure 1:**
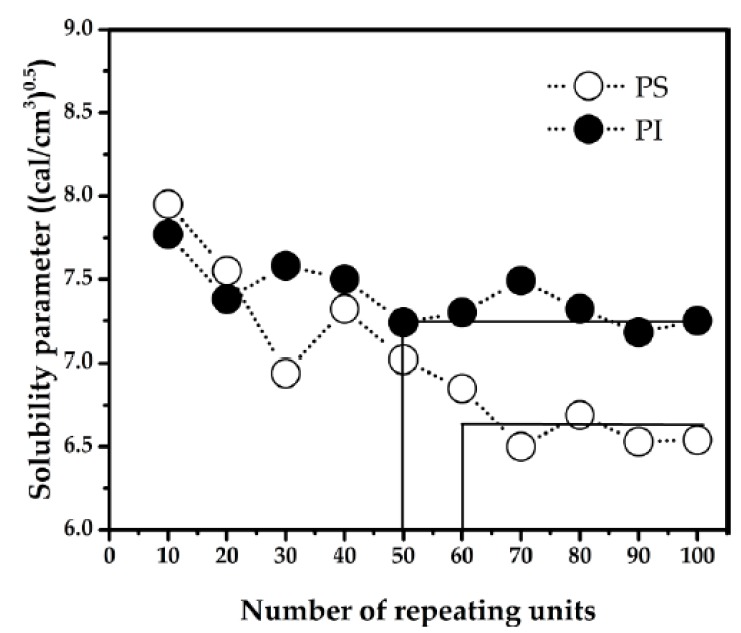
Solubility parameter versus number of repeating units at 393 K for polystyrene (PS) and polyisoprene (PI).

From MD simulation, MCL of isoprene and styrene were determined. The dependence of solubility parameter value on number of repeating units of each polymer was shown in Figure 1. The MCL was estimated from the graph between solubility parameter and number of repeating units. At 393 K, the solubility parameter of isoprene approaches a steady value when the number of repeating units reaches 50 units. On the other hand, the solubility parameter of styrene is nearly constant when the number of repeating units is greater than 60. Hence, the MCL of isoprene and styrene are 50 and 60 units, respectively.

Sub-systems including pure isoprene, pure styrene, and mixed models were simulated. The interaction energy for mesoscopic simulation, called MesoDyn input parameter was calculated from the solubility parameters obtained from these three sub-systems. An effective *χ* parameter or effective Flory-Huggins segment–segment interaction parameter was calculated by using a mixing energy ($\Delta E_{\mathrm{mix}}$) according to the equation below:

(1) $$\chi \;=\;\left(\frac{\Delta E_{\mathrm{mix}}}{\mathrm{RT}}\right)V_{\mathrm{ref}},$$

where, $V_{\mathrm{ref}}$ is the reference volume, R is the molar gas constant (8.314 J/mol·K) and T is the simulation temperature. Then, the $\Delta E_{\mathrm{mix}}$ can be calculated following the equation:

(2) $$\Delta E_{\mathrm{mix}}\;=\;\phi_{\mathrm{PS}}{\left(\frac{E_{\mathrm{coh}}}{V}\right)}_{\mathrm{pure}}+\;\;\phi_{\mathrm{PI}}{\left(\frac{E_{\mathrm{coh}}}{V}\right)}_{\mathrm{pure}}-\;{\left(\frac{E_{\mathrm{coh}}}{V}\right)}_{\mathrm{mixed}},$$

where, ϕ is a volume fraction of each polymer type, the subscripts PS and PI represent polystyrene and polyisoprene, respectively. The subscripts pure and mixed represent the cohesive energy density (*CED*) of pure components and mixing polymers, respectively. The cohesive energy density was converted from solubility parameter (δ). The δ value of three sub-systems was calculated by analyzing the trajectory from MD simulation where *E*_coh_ is the cohesive energy. A relationship between *CED* and δ was demonstrated as follows:

(3) $$\mathrm{CED}\;=\;\frac{E_{\mathrm{coh}}}{V}=\;\delta^{2},$$

The obtained solubility parameter values, MCL, and final densities of each sub-system at three different temperatures demonstrated in Table 1. The solubility parameter and density of each sub-system slightly decreased at high temperature. The increasing of temperature leads to an increase in the system volume due to the expansion of polymer chain.

**Figure 2:**
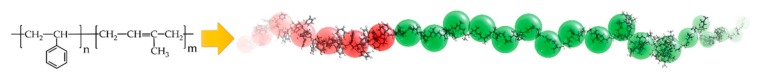
Schematic illustration of a coarse-grained model of PS-*b*-PI copolymer.

Concrete polymer chain length was converted to chain length of Gaussian chain model by using its repeating unit and characteristic ratio which is correlating with each polymer type. The squared end-to-end distance of the Gaussian chain must correspond to the length of the atomistic chain. Each bead in this model is in the same size and represents a segment of the chain up to the polymer persistence length. The chain length of Gaussian chain in a mesoscopic simulation ($N_{\mathrm{meso}}$) was calculated from the following equation [47]:

(4) $$N_{\mathrm{meso}}=\frac{N}{C_{\infty }},$$

where, N is the number of repeating units of polymer equal to polymer molecular weight divided by its monomer molecular weight, and $C_{\infty }$ is the characteristic ratio. The $C_{\infty }$ of PS and PI are 10 and 9, respectively [43]. To represent of 138 PS and 375 PI repeating units, $N_{\mathrm{meso}}$ of PS and PI will be 14 and 42, respectively. Consequently, PS 14 PI 42 was used as a Gaussian chain for mesoscopic simulation. The schematic for converting an actual polymer chain length to a coarse-grained model of PS-*b*-PI copolymer was illustrated in Figure 2. The effective Flory-Huggins segment–segment interaction parameters between distinct beads used in this work at each temperature were listed in Table 2.

### 3.2. Analysis of Phase Morphology and Its Evolution

**Figure 3:**
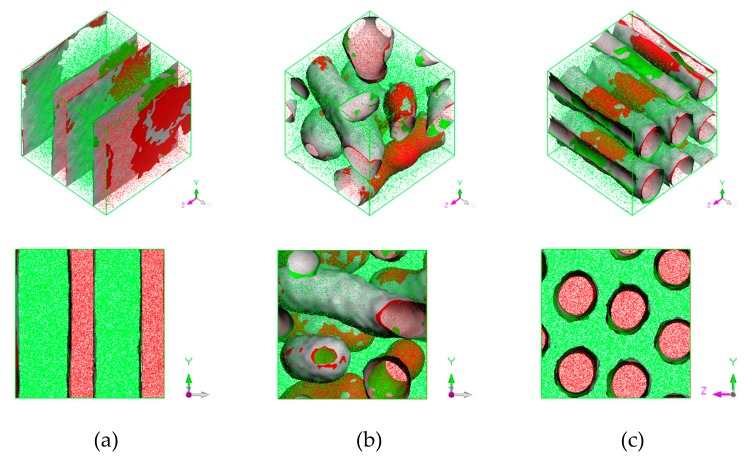
Phase morphology at (**a**) 393 K lamellar, (**b**) 493 K bicontinuous, and (**c**) 533 K hexagonally packed cylinder. Red region represents PS and green region represents PI. Red and green surfaces correspond to PS and PI isosurfaces, respectively, and gray corresponds to interface between them.

It was found that obtained morphologies of PS-*b*-PI copolymer depended on temperature. Three distinct morphologies were obtained, lamellar, bicontinuous, and hexagonally packed cylindrical structures at annealing temperature of 393, 493, and 533 K, respectively. The equilibrium morphologies were shown in Figure 3.

**Figure 4:**
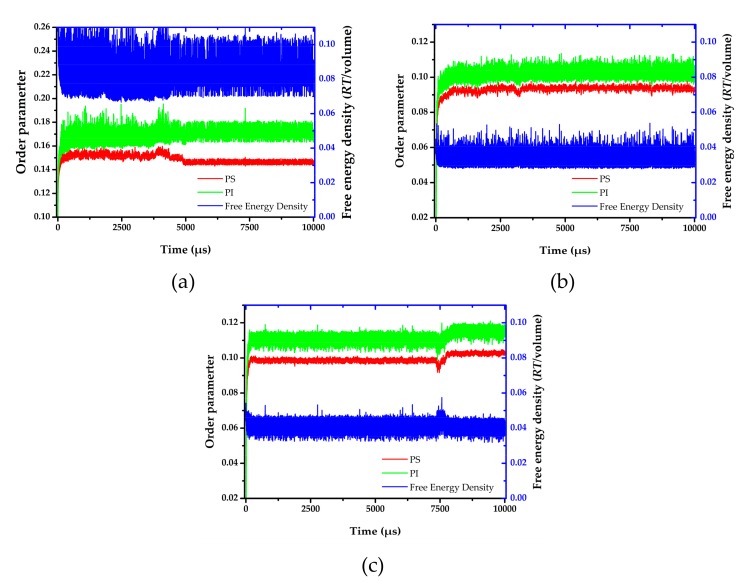
Order parameter and free energy density versus time evolution at (**a**) 393 K, (**b**) 493 K, and (**c**) 533 K.

A characteristic morphology is related with two parameters developed from the simulations, free energy density and order parameter [32]. The free energy density is calculated based on a dynamic mean-field density functional theory. Its tendency can be used for indicating morphology change, morphology at equilibrium state as well as stability of obtained morphology. The order parameter is the mean squared deviation from homogeneity in volume *V*. It is defined as the average volume of the difference between the local density squares and the overall density squared as shown in Equation (5):

(5) $$\mathrm{order}\;\mathrm{parameter}\;=\;\frac{1}{V}\int_{V}\left[\eta_{I}^{2}\left(r\right)-\eta_{I}^{2}\right]\;\mathrm{dr},$$

where, $\eta_{I}$ is dimensionless density (volume fraction) for species I. High order parameter system indicates the phase separation. On the other hand, low value of order parameter indicates the system is more miscibility. The order parameter and free energy density were shown in Figure 4. The order parameter increased in the early stages (I) of the time evolution. In the first 50 µs, the order parameter values and morphology slightly change. After that, the order parameter values swiftly increased, indicating a transition. At this state (II), the same species merged, and the morphology shape rapidly changed before the characteristic morphology was achieved. Finally, the order parameter becomes steady since the aggregated structure reached state (III). Three developed states were also found in a micellar system [48]. In addition, a turning point in order parameter at around 4800 µs and 7400 µs was observed for the annealing system at 393 and 533 K, respectively. This observation indicated a new state (IV). To investigate equilibrium aggregated structure, the distribution of order parameter and free energy density between state (III) and (IV) were determined. The distribution curves of the order parameter and free energy density were illustrated in Figures S1 and S2. The systems annealing at both temperatures show a narrower distribution at the second equilibrium, state (IV).

**Figure 5:**
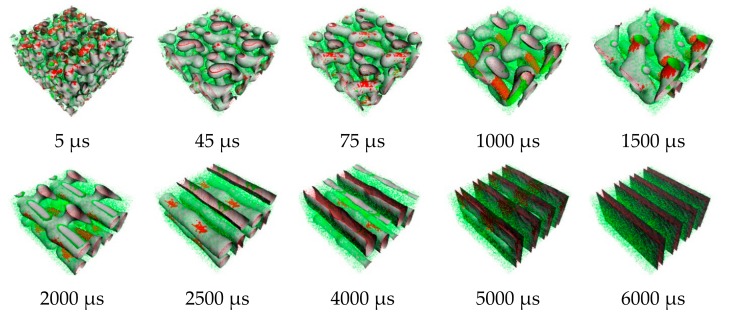
Snapshots of morphology evolution at annealing temperature of 393 K. Red and green regions represent PS and PI, respectively.

**Figure 6:**
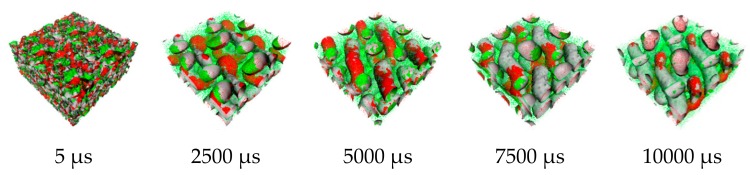
Snapshots of morphology evolution at annealing temperature of 493 K.

**Figure 7:**
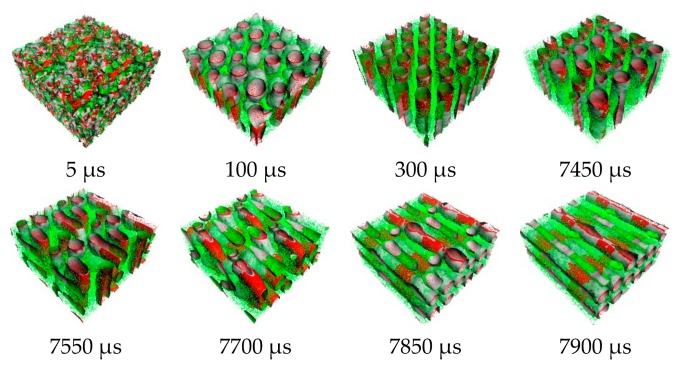
Snapshots of morphology evolution at annealing temperature of 533 K.

From the order parameter in Figure 4a, each system is quite homogenous at the initial state. After that, the aggregated structure immediately changes, and more phase separation occurred. The evolution of PS-*b*-PI copolymer at 393 K was shown in Figure 5. There are fusion and fission processes. These processes were also found in mechanism of spherical micelle formation [11,32]. In fusion process, the polymer in the same species will attach at the center of the aggregation. Then, they will coalesce together starting from their shells to their cores. The larger aggregates can be obtained from this process. The fission process is a reversible process of fusion. The large aggregates can separate to small aggregates in this process. Typically, the fusion process has a chance to produce ordered morphologies leading to various aggregated structures. The PS (red beads), with lower weight ratio, initially merged to form the shapeless core in the first 1500 µs. After that, the morphology approached a pre-cylindrical shape as depicted at 2000 µs. This state was found at first equilibrium with the time evolution was less than 5000 µs as defined as state (III). Beyond 5000 µs, the cylindrical morphology slowly changed into lamellar structure until it reached second equilibrium, state (IV). At the second equilibrium, free energy density of system was converged due to narrower fluctuation. The order parameter of PI increased, while PS decreased due to the morphology arrangement. Over 5000 µs, the red beads adhered together and plane-shape isosurface between distinct polymers were formed. This indicated that the homogeneity of red beads was increased. On the other hand, the green beads separated, indicating higher phase separation. As a result, the order parameter for red beads decrease while that for green beads increase. The previous studies also reported the evolution of a lamellar morphologies via a similar transition pathway [49,50]. The reported metastable morphologies can also be found in our simulations. From Figure 4b, the order parameter and free energy density for PS-*b*-PI copolymer at annealing temperature of 493 K was illustrated. The bicontinuous morphology was obtained at this annealing temperature, its morphology evolution was shown in Figure 6. The bicontinuous structure was slowly formed until the complete bicontinuous morphology was obtained at the equilibrium state. At 533 K, this BCP develops hexagonally packed cylinders. The order parameter and free energy density was shown in Figure 4c. Two equilibrium states, state (III) and (IV), were observed in this simulation comparable to the system annealed at 393 K. However, the tendency of order parameter values was slightly different. Both order parameter values of each polymer type annealed at 533 K increased when a system reached the state (IV). In this case, the change of order parameter related to a rearrangement of polymer chains in the system. While the direction of hexagonally packed cylinders changed, the obtained morphology was considered as the same aggregated structure because the system was studied in a cubic simulation box. The phase aggregation of hexagonally packed cylindrical morphology at 533 K with a time evolution is shown in Figure 7. The spherical and cylindrical morphologies rapidly formed within 0.12 µs due to short lifetime as also reported by Li et al. using a PS 5 PI 5 model [51]. Gyroidal and irregular cylindrical morphologies with longer lifetime were observed in our simulation. The hexagonally packed cylindrical morphology reached equilibrium state within 7900 µs of simulation time.

Three different types of morphologies were obtained in this work. There are lamellar, bicontinuous, and hexagonally packed cylindrical morphologies at 393, 493, and 533 K, respectively. The morphologies agreed well with experimental observation. The experimental data was reported by Khandpur et al. [5]. They reported that lamellar, bicontinuous, and hexagonally packed cylinder were obtained with the same architecture of diblock copolymer, consisted of 138 and 375 actual units of PS and PI, respectively. A volume fraction of PI was equal to 0.675, approximately. They also found that three morphologies were observed when changing annealing temperature and then quenching in liquid nitrogen.

## 4. Conclusions

The theoretical study using a combination of MD and mesoscopic DDF simulation techniques allows us to describe the aggregation behavior of PS-*b*-PI copolymers. The phase behavior and dynamic evolution process was successfully explored. By varying an annealing temperature, the calculated effective Flory-Huggins segment–segment interaction (χ) parameters at 393, 493, and 533 K were 2.2, 0.9, and 1.0, respectively. The increase of the temperature leads to the decrease of effective χ parameter calculated using the solubility parameter from MD simulations.

Three equilibrium morphologies were observed including lamellae, bicontinuous structure, and hexagonally packed cylinders sorting from low to high annealing temperature. The stability of each equilibrium morphology was confirmed by analyzing the tendency of order parameter and free energy density developed from the simulations. Our observed morphologies are consistent with the experimental findings by Khandpur et al. [5] who studied the morphology change in PS-*b*-PI copolymer between composition range from 0.24 to 0.72 polystyrene volume fraction. According to the validity of our approach, the computational protocol presented in this study will be useful to design this BCP with a desired morphology. In addition, our results indicated that various well-defined morphologies can be formed during structural evolution. The simulations successfully reveal how different microstructures of PS can alter its miscibility with PI at different temperatures.

## Figures and Tables

**Table 1:** Solubility parameter, minimum chain length of repeating unit of each polymer (MCL), and density of pure PI, pure PS and mixed systems at different temperatures.

Parameter		Temperature (K)		
		393	493	533
**Solubility parameter** **(cal cm^3^)**	Pure PI	7.22 ± 0.042	6.54 ± 0.044	6.33 ± 0.042
	Pure PS	6.33 ± 0.054	6.19 ± 0.049	6.00 ± 0.065
	Mixed	7.04 ± 0.027	6.69 ± 0.034	6.57 ± 0.036
**MCL** **(units)**	Pure PI	50	40	30
	Pure PS	60	50	30
**Density** **(g cm^3^)**	Pure PI	0.8650	0.8112	0.7868
	Pure PS	0.9993	0.9782	0.9538
	Mixed	0.9163	0.8707	0.8514

**Table 2:** Effective Flory-Huggins segment–segment interaction parameters between distinct beads at different temperatures.

Temperature (K)	Effective χ Parameter
393	2.2
493	0.9
533	1.0

## Supplementary Materials

The following are available online at https://www.mdpi.com/2073-4360/11/6/1008/s1, Figure S1: Distribution of order parameter for (a) PS, (b) PI, and (c) free energy density at 393 K, Figure S2: Distribution of order parameter for (a) PS, (b) PI, and (c) free energy density at 533 K.
