# Supplementary material for: Coarse-Grained Modelling and Temperature Effect on the Morphology of PS-b-PI Copolymer
Source: Polymers (Basel). 2019 Jun 6;11(6):1008. doi: 10.3390/polym11061008 (PMC6630459; doi:10.3390/polym11061008)
Supplement: Supplementary file 1 [file polymers-11-01008-s001.pdf]

Supplementary Materials

Coarse-Grained Modelling and Temperature Effect on the Morphology of PS-*b*-PI Copolymer

Natthiti Chiangraeng [1,2], Vannajan Sanghiran Lee [3] and Piyarat Nimmanpipug [1,*]

[1] Department of Chemistry, Faculty of Science and Center of Excellence for Innovation in Analytical Science and Technology, Chiang Mai University, Chiang Mai 50200, Thailand; natthiti.c@gmail.com

[2] Doctor of Philosophy Program in Chemistry, Faculty of Science, Chiang Mai University, Chiang Mai 50200, Thailand

[3] Department of Chemistry, Faculty of Science, University of Malaya, Kuala Lumpur 50603, Malaysia; vannajan@@um.edu.my

* Correspondence: piyarat.n@cmu.ac.th; Tel.: +66-5394-3344; Fax: +66-5389-2277

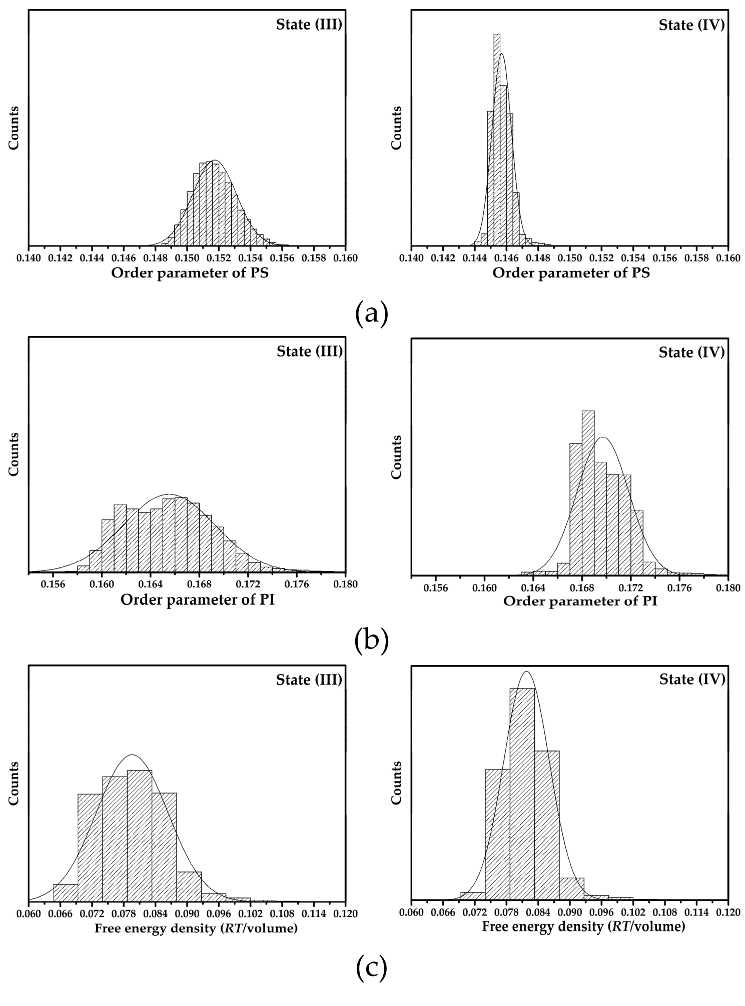

Figure S1. Distribution of order parameter for (a) PS, (b) PI, and (c) free energy density at 393 K.

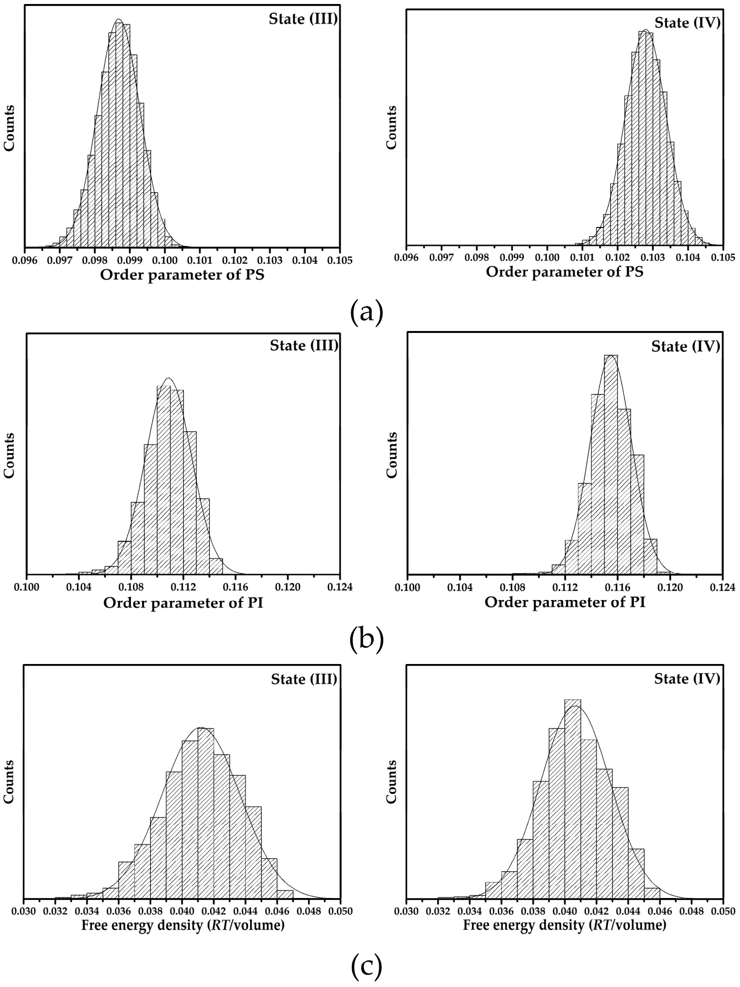

Figure S2. Distribution of order parameter for (a) PS, (b) PI, and (c) free energy density at 533 K.
